# Male reproductive traits of full-sibs of different age classes in three-spined sticklebacks (*Gasterosteus aculeatus*)

**DOI:** 10.1186/2193-1801-2-175

**Published:** 2013-04-20

**Authors:** Marion Mehlis, Theo CM Bakker

**Affiliations:** Institute for Evolutionary Biology and Ecology, University of Bonn, An der Immenburg 1, D-53121 Bonn, Germany

**Keywords:** Ageing, Sperm competition, Sexual selection, Oxidative stress, Carotenoids, Testicular melanisation

## Abstract

The process of ageing is associated with negative effects of mutations acting late in life, which range from those affecting cells to those affecting the whole organism. In many animal taxa, the deterioration of the phenotype with age also affects traits such as males’ primary and secondary sexual characteristics. In three-spined sticklebacks (*Gasterosteus aculeatus*), males usually reproduce at one or two years of age. To see whether sexual selection has the potential to differ between young and old males, full-sib brothers of different age classes were compared, which were bred and raised under standardised laboratory conditions. During two simulated, successive breeding seasons males were allowed to build their nest in single tanks either in the first (“young males”) or in the second (“old males”) breeding season. A comparison of reproductively active brothers of the first and second breeding season showed that older males produce more but smaller sperm, which might be of lower quality. The fact that older males stored more sperm is size dependent as the results show that larger males possess a greater absolute testis mass, which is inextricably linked to sperm number. However, independent of body size, old males had a lower intensity of red/orange and UV breeding coloration as well as a reduced testis melanisation, which might have consequences in female mate choice and sperm competition.

## Background

More than 300 different theories on the proximate and ultimate causes of ageing (Medvedev 1990) have been proposed over the past six decades (for reviews see Hughes and Reynolds 2005 ; Rose et al. 2008 ; Milewski 2010 ; Johnson and Gemmel 2012). Ultimate causes investigate the tension between longevity and reproduction. For example, Medawar (“mutation accumulation theory”: Medawar 1952) postulates that due to natural selection (e.g. predation) individuals within a population do not live long enough to accumulate deleterious mutations that would reduce fitness late in life. Furthermore, Williams (“antagonistic pleiotropy”: Williams 1957) reveals that some genes, which are responsible for an increased reproductive investment when being young, contribute to a lower rate of investment in maintenance and reproduction later in life. Researchers interested in proximate causes focus on mechanisms such as the accumulation of damage to cells and cell function over time caused by free radicals produced during cellular metabolism ("oxidative stress": Harman 1956). To date there is no universal theory of ageing, possibly because the process itself is multicausal (see Holliday 2006).

Theoretical considerations aside, there has recently been considerable interest in the effects of ageing on sexually selected traits. For example, two indicators of male sexual attractiveness, tail length in barn swallows () (Balbontín et al. *Hirundo rustica*^2011^) and foot colour in blue-footed boobies () (Velando et al. *Sula nebouxii*^2011^) were shown to decrease with age. In many animal taxa, ageing has effects on reproductive success, which may be negative (e.g. Dean et al. 2010) as well as positive (e.g. DuVal 2012). In some cases, males of intermediate age were most successful (e.g. Raveh et al. 2010). Also in humans (*Homo sapiens*), testicular volume was significantly different among age classes, with men between 30–60 years showing the greatest testicular volume (Yang et al. 2011). If old males are of lower quality, females will discriminate against them (Beck and Promislow 2007). This exactly happened in the house mouse (*Mus musculus domesticus*); old males and their scent were less attractive to females (Garratt et al. 2011). Similarly, in the field cricket (*Gryllus bimaculatus*), females had a strong preference for the songs of young males (Verburgt et al. 2011). On the other hand, in *Drosophila bipectinata* females preferred to mate with older males, presumably due to the fact that old males mated faster, copulated longer and inseminated more (Somashekar and Krishna 2011). In guppies (*Poecilia reticulata*) old males produced longer but slower sperm and they had larger reserves of strippable sperm when compared to young males (Gasparini et al. 2010). However, artificial insemination did not reveal any effect of age on sperm competition success (Gasparini et al. 2010). Overall, a number of studies investigated the influence of ageing on reproductive traits resulting in contradictory findings, which might partly be explained by the different breeding systems of the study animals (see Johnson and Gemmel 2012 for an overview).

The three-spined stickleback (*Gasterosteus aculeatus*) is a good model system to study the influences of male age on reproductive traits for several reasons. Depending upon location, sticklebacks breed from April to August. Individuals normally reach reproductive age in their second (one-year-old) or third summer (two-year-old) and die after one or two breeding seasons (but see Gambling and Reimchen 2012 for a report of some populations in which individuals live for six years or more). During the breeding season males establish territories in the shallow shore region and build a nest composed of filamentous algae. Furthermore, males usually develop a conspicuous red/orange coloration in the cheek region, court many females and provide all of the parental care (Wootton 1976). The intensity of male breeding coloration is one of the key determinants of female mate-choice decisions (e.g. McLennan and McPhail 1990 ; Bakker and Milinski 1993). Once a female has made her choice, she pushes her way into the male’s nest, deposits her eggs and then leaves as the territorial male enters the nest behind her to fertilise the clutch. Female choice can be circumvented to an extent by sneaking behaviour, in which an intruding male dashes through the nest to deposit sperm immediately before or after the territory holder. This is a common behavioural tactic of territorial males in many populations (e.g. Largiadèr et al. 2001), indicating that sperm competition is an important part of the stickleback breeding system. A recent study revealed that competitive males, which had succeeded in establishing a territory and attracting ripe females, performed most of the sneak attempts (Candolin and Vlieger 2013).

In the three-spined stickleback spermatogenesis only occurs during the short photoperiods preceding the breeding season (Borg 1982), resulting in a larger number of sperm stored in testes of virgin males (e.g. early spring) compared to those of multiply mated males (e.g. late summer) (Zbinden et al. 2001). As stickleback males are sperm limited over the course of one breeding season, careful sperm allocation is a critical fitness component (Zbinden et al. 2003 , 2004). Overall then, both the intensity of males’ breeding coloration (Bakker and Milinski 1993) and gonadal investment (Cubillos and Guderley 2000) have a positive influence on reproductive success. Hence, on the basis of stickleback males’ breeding behaviour and the different theories of ageing it was investigated whether age has a negative influence on sexually selected reproductive traits (breeding coloration, testis and sperm traits) by using full-sib brothers of different age classes.

## Results

### Statistical analyses

The R 2.9.1 statistical package was used for analyses. All given p-values are based on two-tailed tests. For analyses different linear mixed effect models (“lme”) were constructed using males’ reproductive traits (breeding coloration, testis and sperm traits) as dependent variables and male age (young or old) as explanatory variable (Table 1). Furthermore, young and old males originated from the same family (see Methods) implying that they are not independent from each other on a genetic basis. Thus, in all models family identity was included as random factor and never removed to control for possible ancestral influences. Tests of significance were based on likelihood-ratio tests (“LRT”). The use of “lme” requires that the dependent variable is normally distributed. To achieve normal distributions of the residuals of the best explaining models according to Kolmogorov-Smirnov tests, some data were logarithmically transformed (body mass, absolute and relative testis mass, sperm number, total brightness (dorsal region) and sperm tail length), square-root transformed (relative red area of the breeding coloration in the cheek region), exponentially transformed (red/orange hue, testis melanisation (L*_total_)) or reciprocally transformed (total brightness (cheek region)). For a detailed description of all measured reproductive traits see Methods.

**Table 1:** **Listing of all conducted linear mixed effect models “lme”**

Dependent variables	Explanatory variable male age		Explanatory variable body size		AIC male age	AIC body size	Sample size (N)	
	χ^2^	p-value	χ^2^	p-value			Young	Old
Body size [cm]	47.842	<0.001	/	/	61.707	/	60	101
Body mass [g]	39.942	<0.001	/	/	−90.424	/	60	101
Body condition	0.448	0.503	/	/	−315.952	/	60	101
Red area [%]	0.192	0.662	5.245	0.022	607.296	602.243	59	101
Carotenoid chroma (A)	5.312	0.021	4.447	0.035	−59.083	-58.218	59	99
UV chroma (A)	11.091	<0.001	0.855	0.355	−538.148	−527.912	59	99
Red/orange hue (A) [nm]	3.985	0.046	1.269	0.260	43316.630	43319.350	59	99
Total brightness (A)	8.210	0.004	6.903	0.009	−1240.811	−1239.504	59	99
Total brightness (R)	16.374	<0.001	1.595	0.207	262.555	277.334	59	101
Absolute testis mass [g]	54.517	<0.001	76.359	<0.001	−452.293	−474.135	60	96
Relative testis mass (GSI)	20.887	<0.001	3.304	0.069	45.989	63.572	60	96
Testis melanisation (L*_total_)	64.591	<0.001	19.391	<0.001	1955.718	2008.869	60	96
Sperm number	4.256	0.039	11.441	<0.001	223.120	207.984	59	99
Head length [μm]	73.952	<0.001	15.977	<0.001	−277.861	−219.886	60	22
Tail length [μm]	11.816	<0.001	6.773	0.009	−163.597	−158.555	60	22
Head width [μm]	53.729	<0.001	9.646	0.002	−303.683	−259.599	60	22
Mid-piece width [μm]	4.268	0.039	0.676	0.411	−271.444	−267.852	60	22
Mid-piece volume [μm^3^]	9.003	0.003	3.934	0.047	−298.985	−293.916	60	22
Mid-piece length [μm]	2.537	0.111	2.369	0.124	−225.356	−225.187	60	22
Head to tail length ratio	0.052	0.819	0.036	0.849	−540.046	-540.030	60	22

(A) colour measurements were made directly below the eye (breeding coloration).

(R) measurements were taken at the dorsum below the first dorsal spine.

(GSI) gonadosomatic index after de Vlaming et al. (1982).

(L*_total_) testis brightness (see Mehlis et al. 2012 for details).

In all models family identity was included as random factor and never removed to control for possible ancestral influences. The model with the lowest AIC (Akaike's information criterion) value represents the best approximating model.

### Body traits

Young (N_young_ = 60) and old males (N_old_ = 101) differed significantly in body size (“lme”, χ^2^ = 47.842, P < 0.001) and mass (“lme”, χ^2^ = 39.942, P < 0.001), showing that older males were on average larger and heavier than younger ones (Table 2). Hence, whether age or size affected the observed results (see below) is hard to distinguish due to the fact that age and body size are not statistically independent from each other. Accordingly, one has to mention that body size of the used individuals was determined by age as fish grow throughout their life time. It should be valid to use the base of differences (age) in the statistical analyses in order to find differences in reproductive traits and discuss them in an evolutionary context. Nevertheless, all models (Table 1) were run again but instead of using male age body size was included as explanatory variable. The results of these models are also listed in Table 1. Akaike’s information criterion (AIC) was used for model comparison (male age or body size as explanatory variable); the model with the lowest AIC value represents the best approximating model (Symonds and Moussalli 2011). The results showed that, apart from the relative red area of males’ breeding coloration, absolute testis mass and sperm number, male age is more appropriate in explaining the observed results than body size (Table 1). Thus, only the statistically relevant results concerning male body size are described in detail and discussed later on. Moreover, young and old males did not differ significantly in physical conditions (“lme”, χ^2^ = 0.448, P = 0.503), indicating that they had the same pre-condition during life time (Table 2).

**Table 2:** **Descriptive statistics (median, first and third quartile) of body traits, breeding coloration variables and testis and sperm traits of young and old males used in the analyses**

	Young			Old		
	Median	1. Quartile	3. Quartile	Median	1. Quartile	3. Quartile
Body size [cm]	4.90	4.70	5.10	5.20	4.95	5.40
Body mass [g]	1.562	1.372	1.712	1.812	1.617	2.031
Body condition	1.30	1.25	1.36	1.29	1.24	1.35
Red area [%]	14.84	6.22	27.21	13.74	6.29	21.94
Carotenoid chroma (A)	0.450	0.287	0.538	0.392	0.189	0.501
UV chroma (A)	0.271	0.244	0.291	0.245	0.209	0.273
Red/orange hue (A) [nm]	506.0	502.0	509.5	505.0	501.0	508.5
Total brightness (A)	2701.70	2072.17	3719.65	3267.38	2489.20	4939.09
Total brightness (R)	3075.86	2374.82	4326.12	1963.18	1402.66	3114.35
Absolute testis mass [g]	0.0107	0.0084	0.0140	0.0165	0.0135	0.0202
Relative testis mass (GSI)	0.697	0.585	0.862	0.951	0.731	1.103
Testis melanisation (L*_total_)	39.00	26.88	47.13	60.50	49.38	67.50
Sperm number	9.3_*_10^6^	6.0_*_10^6^	12.2_*_10^6^	10.7_*_10^6^	7.9_*_10^6^	14.4_*_10^6^
Head length [μm]	1.106	1.067	1.128	0.991	0.958	1.011
Tail length [μm]	18.033	16.946	19.280	17.017	16.036	17.600
Head width [μm]	1.076	1.048	1.104	0.999	0.979	1.018
Mid-piece width [μm]	0.782	0.761	0.813	0.766	0.731	0.792
Mid-piece volume [μm^3^]	0.284	0.268	0.311	0.256	0.239	0.281
Mid-piece length [μm]	0.587	0.548	0.614	0.583	0.515	0.621
Head to tail length ratio	0.094	0.089	0.099	0.091	0.087	0.098

(A) colour measurements were made directly below the eye (breeding coloration).

(R) measurements were taken at the dorsum below the first dorsal spine.

(GSI) gonadosomatic index after de Vlaming et al. (1982).

(L*_total_) testis brightness (see Mehlis et al. 2012 for details).

### Breeding coloration

**Figure 1:**
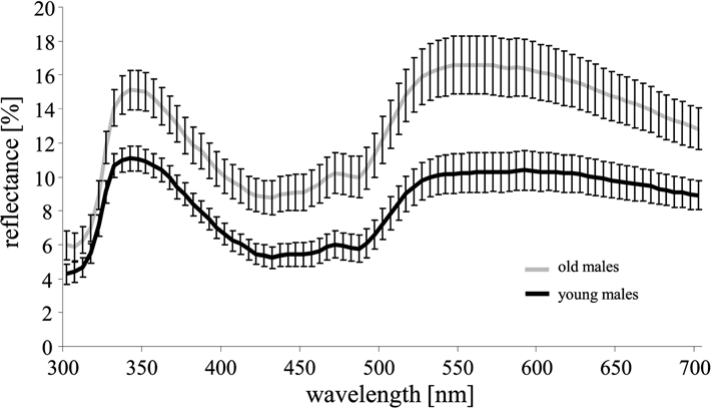
**Representative reflectance spectrum of the carotenoid-based breeding coloration of three-spined stickleback males.** Mean reflectance ± standard error (%) between 300 and 700 nm of the red/orange breeding coloration in the cheek region of reproductively active young (black line) and old (grey line) males. Reflectance was measured relative to a 98% Spectralon white standard.

The relative area of red breeding coloration in the cheek region did not differ significantly between young and old males (N_young_ = 59, N_old_ = 101; “lme”, χ^2^ = 0.192, P = 0.662; Table 2) but it was significantly explained by body size (“lme”, χ^2^ = 5.245, P = 0.022), with larger males showing a significantly greater relative area of the red breeding coloration. However, young and old males differed significantly in carotenoid chroma (N_young_ = 59, N_old_ = 99; “lme”, χ^2^ = 5.312, P = 0.021) as well as in UV chroma (N_young_ = 59, N_old_ = 99; “lme”, χ^2^ = 11.091, P < 0.001), with larger chroma values for young males in both cases (Figure 1; Table 2). Thus, young males had a significantly more saturated coloration in the red/orange spectral region. In addition, the red/orange hue was significantly lower in old males (N_young_ = 59, N_old_ = 99; “lme”, χ^2^ = 3.985, P = 0.046), indicating a more red-shifted hue of the breeding coloration in young males compared to old males (Figure 1; Table 2). Moreover, younger males showed a significantly lower total brightness in the cheek region (N_young_ = 59, N_old_ = 99; “lme”, χ^2^ = 8.210, P = 0.004; Table 2) and a significantly higher total brightness in the dorsal region (N_young_ = 59, N_old_ = 101; “lme”, χ^2^ = 16.374, P < 0.001; Figure 1; Table 2).

### Testis traits

**Figure 2:**
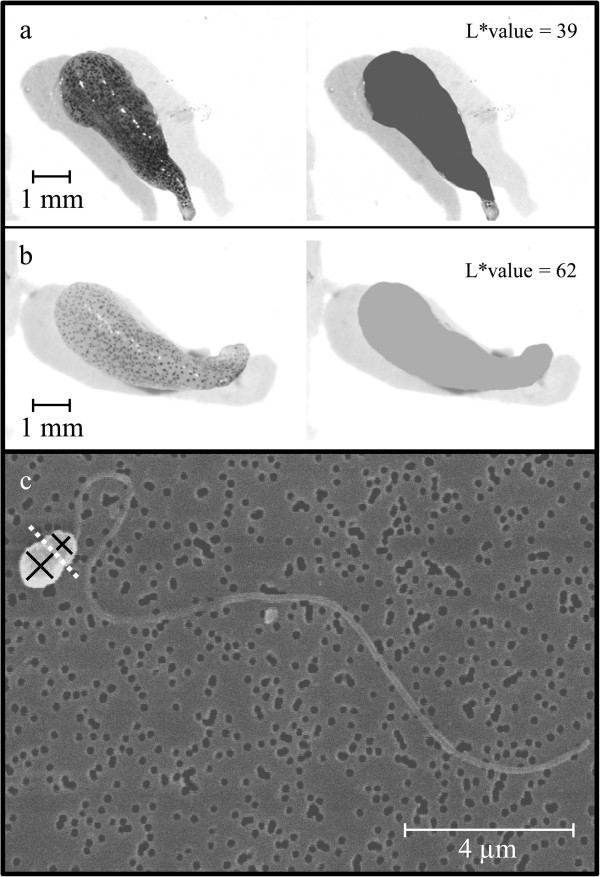
**Representative testes and sperm of three-spined stickleback males.** Testis of a young (**a**) and an old (**b**) male and on the right side the corresponding L*value for testis brightness as a measure of testis melanisation (see Mehlis et al. 2012). (**c**) Sperm head, mid-piece, and tail can be distinguished on the SEM image. The black lines indicate head length and width as well as mid-piece length and width, the dotted white line indicates the boundary between head and mid-piece.

Young (N_young_ = 60) and old males (N_old_ = 96) differed significantly in absolute (“lme”, χ^2^ = 54.517, P < 0.001) and relative testis mass (“lme”, χ^2^ = 20.887, P < 0.001) as well as in total testis brightness (“lme”, χ^2^ = 64.591, P < 0.001), which is a measure of testicular melanisation (see Mehlis et al. 2012). While absolute and relative testis mass were both significantly higher in older males, the results show that testis melanisation was significantly lower in young males meaning that the testes of old males were less pigmented (Figure 2a-b; Table 2). However, it has to be mentioned that differences in absolute testis mass were better explained by differences in body size (lower AIC) than by male age alone, with larger males having significantly heavier testes (“lme”, χ^2^ = 76.359, P < 0.001).

### Sperm traits

Stored sperm number differed significantly between young and old males (N_young_ = 59, N_old_ = 99; “lme”, χ^2^ = 4.256, P = 0.039), with older males having significantly more sperm (Table 2). However, the statistical analysis revealed that sperm number was explained in a more appropriate way by male body size (lower AIC) than by male age, with larger males having significantly more sperm (“lme”, χ^2^ = 11.441, P < 0.001). Young (N_young_ = 60) and old males (N_old_ = 22) differed significantly in sperm morphology traits (see Figure 2c), such as head length (“lme”, χ^2^ = 73.952, P < 0.001), tail length (“lme”, χ^2^ = 11.816, P < 0.001), head width (“lme”, χ^2^ = 53.729, P < 0.001), mid-piece width (“lme”, χ^2^ = 4.268, P = 0.039) and mid-piece volume (“lme”, χ^2^ = 9.003, P = 0.003), with young males showing higher values in all of these measures (Table 2). However, sperm of young and old males did not differ significantly in mid-piece length (“lme”, χ^2^ = 2.537, P = 0.111; Table 2) and head to tail length ratio (“lme”, χ^2^ = 0.052, P = 0.819; Table 2).

## Discussion

By comparing reproductive traits of lab-reared full-sib brothers of different ages in their first reproductive season (i.e. first nest building), the present study revealed that an advanced age can have considerable consequences on the expression of reproductive traits in three-spined sticklebacks, which might have an influence in inter- as well as intra-sexual contexts.

In three-spined sticklebacks sperm competition occurs in many populations (e.g. Goldschmidt et al. 1992 ; Jamieson and Colgan 1992 ; Rico et al. 1992 ; Largiadèr et al. 2001 ; Vlieger and Candolin 2009). Furthermore, it is generally accepted that sperm competition is a widespread and powerful selective force affecting sperm quantity and/or sperm quality in such a way to maximise competitive fertilisation success (e.g. Rowe and Pruett-Jones 2011). In a situation in which the risk of sperm competition is high, males should have larger testes and/or a higher number of sperm per ejaculation (Harcourt et al. 1981 ; Stockley et al. 1997) assuming that fertilisation success follows a raffle-principle, so that males with more sperm will have an advantage (see Parker 1990). In the American horseshoe crab (*Limulus polyphemus*) young males ejaculated more total sperm and had a significantly higher sperm concentration than old males (Sasson et al. 2012). In contrast, in the guppy (*Poecilia reticulata*) and in zebrafish (*Danio rerio*), the number of extractable sperm was higher in old males (Gasparini et al. 2010 ; Kanuga et al. 2011). In the zebrafish study the authors argued that this might be a consequence of fewer successful breeding attempts of old zebrafish males. In three-spined sticklebacks, the number of stored sperm is significantly positively correlated with testis mass (Zbinden et al. 2001), and gonad mass is related to reproductive success (Cubillos and Guderley 2000) as is body size (Kraak et al. 1999), showing the adaptive importance of these variables. In the present study, larger (older) stickleback males also had significantly more stored sperm as well as a higher absolute testis mass. It is hard to distinguish whether size or age affected the observed results, due to the fact that age and body size are not statistically independent from each other and especially in fish they are inextricably linked. However, all stickleback males used here were virgin as they had no opportunity to release sperm during their entire life span under laboratory conditions. In the rainbow trout (*Oncorhynchus mykiss*) it is known that unused sperm are reabsorbed during the post-spawning period, just before the next cycle of spermatogenesis begins (Billard and Takashima 1983), which could also be assumed for sticklebacks as in this species sperm production undergoes an annual cycle (Borg 1982). Furthermore, in a previous study it was stated that at the end of the breeding season sticklebacks testes were drastically reorganised (Schneider 1969), which is supported by the fact that testis mass declines after the breeding season (e.g. Hoffmann et al. 2012). Hence, it is likely that the sperm, which were found in the two-year-old males, were solely produced during the second winter period, leading to the interpretation that larger old males produced both absolutely and relatively more sperm.

If a male produces more gametes, these sperm will be proportionally smaller, assuming that a fixed “resource budget” is available (Parker 1982) and in the present study sperm dimensions were indeed larger in young males. Sperm morphology traits, such as sperm size, are known to be good proxies of reproductive performance in several animal species (Gomendio and Roldan 1991). For example, some studies have shown that sperm swimming ability was positively correlated with either head length, tail length or the sum of both (Pitcher et al. 2007 ; Gomendio and Roldan 2008 ; Fitzpatrick et al. 2010). However, this topic is controversial (see Snook 2005 for an overview) and many studies found no relationship between sperm morphology traits and sperm swimming performance (e.g. Gage et al. 2002 ; Locatello et al. 2007). Nevertheless, there is evidence that sperm motility is associated with mid-piece volume (Anderson and Dixson 2002) and the evolution of sperm mid-piece volume has been affected by selection pressures resulting from sperm competition (Anderson et al. 2005); the higher the mid-piece volume the higher the mitochondrial loading and thus flagellum beating frequency (Cardullo and Baltz 1991). The results of the present study show that sperm size and mid-piece volume were significantly larger in young males, suggesting higher quality sperm, as in sticklebacks fertilisation success is predicted by longer tail length, greater mid-piece volume and smaller head to tail length ratio (TCMB et al. unpublished data). Young and old males did however not differ significantly in head to flagellum length ratio, another proxy for sperm motility (Humphries et al. 2008), leaving this issue open for further investigations.

Sperm are known to be highly prone to oxidative stress (e.g. Aitken and Baker 2006). Helfenstein et al. (2010) reported the first experimental evidence that development of carotenoid-based ornaments and sperm quality may be linked through oxidative stress. Carotenoid-based conspicuous yellow, orange and red colour patterns are widespread in many vertebrate species (e.g. Hill et al. 2002). The colour variables for the stickleback cheek region that were measured in the present study indicate a stronger deposition of carotenoids in terms of a more chromatic and darker breeding coloration in younger males (Gomez et al. 2011) as an indicator of higher individual quality compared to older males. In sticklebacks, the intensity of the red/orange breeding coloration is one of the key determinants of female mate-choice decisions (e.g. McLennan and McPhail 1990 ; Bakker and Milinski 1993). If carotenoid availability is limited, these pigments will be traded off between fitness components such as sexual ornamentation and immunoresponsive capacities (Lozano 1994), thereby maintaining the honesty of carotenoid-based signals (e.g. Blount et al. 2003 ; but see Navara and Hill 2003 spec. larvae that contain a.o. lutein (Czeczuga ). In the laboratory, both young and old males were fed *Chironomus*^1970^), a major carotenoid for the development of males’ breeding coloration (Wedekind et al. 1998 . Since animals cannot synthesise carotenoids *de novo*(Goodwin 1984), the given food supply was the only carotenoid source for the males. The less developed breeding coloration of older males might imply that old males’ ability to cope with oxidative stress may be reduced, thus supporting the oxidative stress ageing theory as suggested by Harman (1956).

Testis melanisation occurs in many animal taxa, for example in fish (Louiz et al. 2009), amphibians (Zieri et al. 2007), reptiles (Guillette et al. 1983), birds (Galván et al. 2011), and mammals (Poole and Lawton 2009). In birds, for example, testicular melanisation has been evolved in species with high rates of accumulated mitochondrial mutations and has been supposed to be an adaptive response related to the protective capacity of melanin against oxidative stress (Galván et al. 2011). The current study showed that the testes of older stickleback males were less pigmented, indicating that younger males might be able to handle oxidative stress better than their older brothers (but see Andziak et al. 2006). At the same time younger males were less pigmented in the dorsal region. This may indicate a trade-off between the deposition of melanin pigments in skin and testis tissue. Such a correlation between the proportion of plumage coloured by melanin and occurrence of testicular melanisation has not been found in birds (Galván et al. 2011). However, the precise function and relevance of testis melanisation in sticklebacks is unknown and requires further investigation.

Older males were significantly larger and heavier than their younger brothers. This is not surprising due to the fact that fish have indeterminate growth and also in sticklebacks age is largely confounded with body size (Allen and Wootton 1982). Furthermore, in three-spined sticklebacks, larger males are more aggressive (Mehlis et al. 2010), have an advantage in dominance fights (Rowland 1989), and females find larger males more attractive possibly due to their higher paternal quality and/or territorial quality (Kraak et al. 1999). However, females also prefer more intensely red-coloured stickleback males (Bakker and Milinski 1993 ; Kraak et al. 1999 ; Künzler and Bakker 2001) and the results of the present study show that the relative area of red breeding coloration was greater in large males. It remains unclear how the attractiveness in terms of breeding coloration (higher saturated coloration and hue in the red/orange spectral region in young males but greater area of red breeding coloration in large old males) is traded off against body size, and thus whether younger males have the edge over their older brothers.

## Conclusions

To conclude, the results revealed that advanced age can have beneficial as well as detrimental effects on an individual male stickleback’s reproductive traits. Older males were larger (dominance advantage) and had more sperm than their younger brothers. They might be, however, at a disadvantage in terms of mate acquisition (less intense nuptial signal) and fertilisation ability (lower quality sperm). The less developed nuptial colour, testis melanisation and sperm quality could be the result of oxidative stress to cells and tissues accumulating over time (Harman 1956). However, one has to mention that further investigations are needed to confirm that the results of this explanatory study are really important under natural conditions, especially when stickleback males are confronted with sperm competition and whether this influences males’ fertilisation abilities and reproductive success. Furthermore, given the damaging effects of progressive ageing, it is interesting to note that most males from the study population do not live long enough to reach a second reproductive phase in the field. Taken together, these observations support ultimate ageing theories suggesting that an age-related decrease in fitness might select for an increased reproductive investment early in life (Medawar 1952 ; Williams 1957).

## Methods

### Experimental subjects

About 500 three-spined sticklebacks from an anadromous, genetically heterogeneous population (Heckel et al. 2002) were caught during their spring migration in April 2008 on the island of Texel, the Netherlands. Sticklebacks were purchased from a commercial fisherman, who has the permission to catch the fish. Within 5–6 hours fish were transported in large boxes (half filled with sea- and tap-water) to the Institute for Evolutionary Biology and Ecology in Bonn, Germany, where they were kept together in a large outside-tank (750 l), with air ventilation and a constant supply of tap-water at a flow rate of 3l min^-1^ and fed with red mosquito larvae (*Chironomus* spec.). The probability that individuals from the used study population die after their first breeding season seems to be high due to two reasons. First, the frequency distribution of standard lengths of the sampled fish during spring migration was single-peaked (MM personal observation). Second, a previous study did not find any large individuals during autumn migration from the breeding sites to the sea (van Mullem and van der Vlugt 1964). Nevertheless, under laboratory conditions it is common that these fish live 30 months or even longer and still reproduce (e.g. Mehlis et al. 2008 ; Mehlis et al. 2012).

To achieve different family groups, a randomly chosen male was allowed to spawn with a randomly chosen female, which were transferred to the laboratory with simulated summer conditions (day length 16L:8D, temperature 17 ± 1°C) for reproduction; thereby 40 clutches were produced in June 2008. Parents were only used once to avoid pseudoreplication. Thus, males used in this study originated from the F1 generation of randomly crossed wild-caught sticklebacks. To exclude paternal effects clutches were removed from the males’ tanks 2 h after fertilisation and split into two full-sib groups. Hatched fry were fed daily with *Artemia* nauplii during the first month of age and juveniles/adults were fed with red mosquito larvae (*Chironomus* spec.) *in excess* later on. At an age of three weeks group size was reduced to 50 individuals, which were kept in holding tanks measuring 30 cm × 20 cm × 20 cm (length × width × height). Twenty weeks later, fish were transferred to holding tanks measuring 50 cm × 30 cm × 30 cm. Groups that consisted of more than 20 individuals were split again (eleven groups originating from eight families) and families that consisted of fewer than 20 individuals were fused again (15 families). At this time point all holding tanks were placed in an air-conditioned room under standardised winter light-regime (8L:16D, 17 ± 1°C). In sticklebacks, sexual maturation is stimulated by long photoperiods (Borg et al. 2004). Hence, to start the breeding season the light regime was changed to summer conditions in May 2009 (16L:8D, 17 ± 1°C), which lasted five months and followed by a second simulated winter period. At an age of 20 months fish were put under summer conditions again.

During both simulated breeding seasons randomly chosen males that showed signs of nuptial coloration in their holding tanks were individually isolated in separate tanks (30 cm × 20 cm × 20 cm) for nest building. It was ensured that all males used were virgin, because they had no possibility to build a nest in their holding tank and they were not paired to any female in their nesting tank. Collecting data such as total testis mass and sperm count (see below) is invasive; hence it was not possible to measure these traits for one male through time. In addition, only males that had built a nest were used, which ensures that they were reproductively active. In total, 161 males originating from 32 families built a nest. This included 60 males from 32 families, which were allowed to build nests in their first summer (“young males”) and 101 males from 22 families, which were not allowed to build nests until the following summer (“old males”). During both simulated breeding seasons, data collection for young and old males followed the same pattern, which is subsequently explained in detail.

### Measurement of body traits

Males’ body size (S) and mass (M) were determined and afterwards their body condition (BC) was calculated following Bolger and Connolly (1989; BC = 100 * M / S^3^). Body measures were taken before isolating the males and shortly before euthanising them (see below). Hence, for each male both values were averaged for further analyses.

### Measurement of breeding coloration

To determine the red area of male breeding coloration standardised digital still photographs were taken inside a black photo box using a camera (Nikon D70s with a Nikkor micro lens) connected to two flashes (Metz 20 BC6). This is a method frequently used in sticklebacks (e.g. Bakker and Mundwiler 1994 ; Frommen et al. 2008). A black curtain was tightened around the photo box to prevent any stray light from entering the box.

Males that had a nest for two days were stimulated with a receptive female (swollen abdomen filled with eggs) for 15 min. Afterwards, the male was put into a transparent plastic box (9.5 cm × 7 cm × 7 cm) containing some water and the left lateral side, visible through high quality glass (Hoya HMC UV filter), was photographed with an exposure time of 1/125 s and focal aperture of 16. Images were processed using Adobe Photoshop CS4. A white Munsell card (N10) visible on each picture served as white balance. The shape of the fish head (from snout to gills) was marked using the magnetic-lasso-tool and subsequently saved on a white background (1400 × 1000 pixel). To finish the quantification of the red area, images of the fish head were uploaded in Sigma Scan Pro 5.0 and the percentage of red area (threshold hue: 0–40; threshold saturation: 20–100) was determined in relation to the total area of the fish head and the white background.

The expression of male breeding coloration was quantified by using reflectance measurements of the skin, which were taken on average two days after the photograph (median and quartiles: 2, 2, 4). Shortly before reflectance measurements, males were stimulated with a receptive female for 15 min. Reflectance scans were taken with a spectrophotometer (Avantes AVS-USB2000) connected to a deuterium-halogen light source (Avantes DH-2000) for illumination (see Rick et al. 2011 for a detailed description). Measurements were made directly below the eye (A-region, breeding coloration) and at the dorsum below the first dorsal spine (R-region). Data were recorded without changing probe contact using Spectrawin 5.1 (Avantes) and imported into Microsoft Excel. About twenty measurements were taken in succession and averaged for the A-region (median and quartiles: 20, 17, 27) and the R-region (median and quartiles: 18, 16, 19).

The reflectance spectrum of the carotenoid-based breeding coloration of three-spined stickleback males is double-peaked with one peak in the UV-region and an extended plateau at longer wavelengths (Rick and Bakker 2008; Figure 1). Hence, for the A-region the UV chroma (C_UV_ = R_300-400_ / R_300-700_; R = reflectance at a given wavelength) and, as a measure of carotenoid pigmentation (e.g. Blount and Pike 2012), carotenoid chroma (C_caro_ = (R_700_ - R_450_) / R_700_) were calculated. The R_50_ value for the A-region was determined, defined as the wavelength at the halfway reflectance point between the minimum reflectance (400 to 500 nm) and the maximum reflectance (500 to 700 nm). Higher R_50_ values indicate a more red-shifted hue of the measured region (Rick and Bakker 2008 ; Pike et al. 2011). Finally, the total brightness (R_300-700_) was measured for both regions.

### Measurement of testis traits

Directly after reflectance measurements were taken, males were euthanised quickly by decapitation, both testes were removed, placed separately on a tissue paper and weighed to the nearest milligram (OHAUS, Explorer, E11140) so that the relative testis mass (gonadosomatic index, GSI,) could be calculated after de Vlaming et al. (1982; GSI = (TM_total_ / M) * 100, where TM_total_ is the sum of the absolute mass of both testes).

As the testes of three-spined stickleback males are covered with melanophores and there is striking between-male variation in testicular melanisation and the L*value (brightness) is a good indicator of melanophore density (see Figure 2 a-b; Mehlis et al. 2012), the intensity of melanophore pigmentation was quantified using the same method as described in Mehlis et al. (2012). Melanophores are generally thought to protect the testes from deleterious UV light due to their strong light absorbance (Kaidbey et al. 1979 ; Plonka et al. 2009) and/or to protect males’ germ cells from oxidative stress (Galván et al. 2011). Testis brightness was determined for each male’s testis (L*_left_ and L*_right_) and finally averaged (L*_total_).

### Measurements of sperm traits

The measurement of sperm number was solely conducted on the left testis because sperm number of left and right testes are highly correlated in sticklebacks (Bakker et al. 2006). Right testes were used for later SEM-preparation (see below) hence they were stored in an Eppendorf tube containing 500 μl 4% formalin. The left testis was pestled in 200 μl of a non-activating medium (for mixture see Fauvel et al. 1999). Ten microlitres of the sperm suspension were diluted in 190 μl of tap water in order to reduce sperm density. Twelve microlitres of this dilution were filled into an improved Neubauer counting chamber (Labor Optik, 0.0025 mm^2^, depth 0.1 mm). Sperm were counted in 32 equally distributed cells and averaged, so that the total number of sperm was calculated (see Mehlis et al. 2012).

Sperm morphology was determined by scanning electron microscopy (SEM; Figure 2c). The preparation of sperm followed Mortimer (1994) and was adapted for sticklebacks providing highly repeatable measurements of sperm traits (see Mehlis et al. 2012). Sperm variables were measured using 3000–6000 magnified SEM-images (Leitz AMR 1000), which were digitised using DISS (Digital Image Scanning System, Point Electronic GmbH, Halle, Germany) and DIPS (Digital Image Processing System V 2.5.2.1, Point Electronic GmbH, Halle, Germany). Sperm morphology variables (head length (hl), head width (hw), mid-piece length (ml), mid-piece width (mw) and tail length (tl)) were measured only on images that showed intact sperm (with a well visible head and a complete tail; see Figure 2c) using ImageJ. On average 20 sperm were measured per male (median and quartiles: 20, 19, 22).

Unfortunately, it was not possible to measure sperm motility directly because after dissection testes were stored in 4% formalin. However, in stickleback’s fertilisation success is predicted by tail length, mid-piece volume and head to tail length ratio (TCMB et al. unpublished data). Thus, the head to tail length ratio ((hl + ml) / tl) and mid-piece volume (π * (mw / 2)^2^ * ml) were calculated as a proxy of sperm motility being an indicator of sperm quality.
